# Top classic citations in pancreatic cancer research

**DOI:** 10.1186/s12957-016-1061-8

**Published:** 2016-11-29

**Authors:** Qiang Li, Yuan Jiang

**Affiliations:** 1 Department of Emergency Medicine, Second Affiliated Hospital, School of Medicine, Zhejiang University, Hangzhou, 310009 China; 2 Department of Internal Medicine, Children’s Hospital, School of Medicine, Zhejiang University, Hangzhou, 310003 China

**Keywords:** Citation classic, Pancreatic cancer

## Abstract

**Background:**

The number of times that articles are cited by is widely used to evaluate the impact of an article or an individual author has on its scientific community. This bibliometric analysis aimed to explore the top classic citations in pancreatic cancer (PC) research.

**Methods:**

A computerized literature search was conducted using the database, the Science Citation Index Expanded. The top 100 highly cited articles were included and further analyzed.

**Results:**

The most cited article had 3,032 citations, with a mean of 626 citations per paper. These highly cited articles were published in 37 journals, led by *Cancer Research* (15 articles). Of the 100 articles, 40 were observational studies, 36 dealt with basic science, and 14 were randomized controlled trials. These articles came from 11 countries, with the USA contributing 79 articles. Fifty-one institutions produced these 100 citation classics, led by Johns Hopkins University (20 articles). Twenty-seven persons authored two or more of the top-cited articles, led by Kern SE (6) and Yeo CJ (5).

**Conclusions:**

This analysis of the top highly cited articles allows for the recognition of major advances in PC research and gives a historic perspective on the progress of this specialty of PC research.

## Background

Currently, pancreatic cancer (PC) remains a fatal disease with poor prognosis [1]. In recent decades, significant growth has been seen in the field of pancreatic cancer research. Though these advancements in PC research are widely known by the clinicians, the most important papers are not commonly known and identified. Recently, many specialities and diseases have identified and analyzed their “citation classics” (the articles most highly cited or the articles cited more than 100 times) in their fields [2–6]. Besides, there were also a few journals which published their own citation classics [7, 8]. The purpose of our present study was to identify the top classic citations in PC research.

## Methods

A computerized literature search was conducted using the database, the Science Citation Index Expanded founded by the Institute for Scientific Information (1966–Sep 25, 2015) [9]. The relevant articles were identified by searching using a highly sensitive search strategy, and all phases of the search strategy are shown in Table 1. There was no journal restriction. The top 100 highly cited articles were included for further analysis.

**Table 1:** All phases of the search strategy to identify potential articles

title=(pancreatic or pancreas) AND title=(adenocarcinoma or adenocarcinomas or carcinoma or carcinomas or cancer or cancers or neoplasm or neoplasms or tumor or tumors or malignancy or malignancies)	

The titles and abstracts of the articles would be reviewed to estimate whether they are related to PC research. We analyzed the articles and calculated the data according to the following predefined items: number of citations, publication year, country of origin, institution, journal, publication type (e.g., basic science, observational study), and authorship (only calculating the corresponding, first, and second author).

## Results

The literature search involved 27,413 publications using the Science Citation Index Expanded founded by the Institute for Scientific Information (ISI) (1966–present). The top 100 highly cited articles were showed in Table 2 according to the number of citation. The most frequently cited article received 3032 citations, and the least frequently cited article received 330 citations. Twelve articles received more than 1000 citations. The mean number of citations per paper was 626. We also evaluated the 10-year citations of the paper published before 2006. The most frequently cited article received 1354 citations, and the least frequently cited article received 5 citations. The proportions of 10-year after publication of 1970–1979, 1980–1989, 1990–1999, and 2000–2005 were 20.5, 41.2, 49.8, and 68.0%, respectively (Table 3).

**Table 2:** The top 100 cited articles in pancreas cancer research

Rank	Article	No. of citations
1	Burris HA 3rd, Moore MJ, Andersen J, et al. Improvements in survival and clinical benefit with gemcitabine as first-line therapy for patients with advanced pancreas cancer: a randomized trial. J Clin Oncol. 1997;15(6):2403-2413.	3032
2	Moore MJ, Goldstein D, Hamm J, et al. Erlotinib plus gemcitabine compared with gemcitabine alone in patients with advanced pancreatic cancer: a phase III trial of the National Cancer Institute of Canada Clinical Trials Group. J Clin Oncol. 2007;25(15):1960-1966.	1731
3	Jones S, Zhang X, Parsons DW, et al. Core signaling pathways in human pancreatic cancers revealed by global genomic analyses. Science. 2008;321(5897):1801-1806.	1706
4	Li C, Heidt DG, Dalerba P, et al. Identification of pancreatic cancer stem cells. Cancer Res. 2007;67(3):1030-1037.	1620
5	Almoguera C, Shibata D, Forrester K, et al. Most human carcinomas of the exocrine pancreas contain mutant c-K-ras genes. Cell. 1988;53(4):549-554.	1567
6	Guillemin R, Brazeau P, Böhlen P, et al. Growth hormone-releasing factor from a human pancreatic tumor that caused acromegaly. Science. 1982;218(4572):585-587.	1291
7	Conroy T, Desseigne F, Ychou M, et al. FOLFIRINOX versus gemcitabine for metastatic pancreatic cancer. N Engl J Med. 2011;364(19):1817-1825.	1282
8	Warshaw AL, Fernández-del Castillo C. Pancreatic carcinoma. N Engl J Med. 1992;326(7):455-465.	1254
9	Hermann PC, Huber SL, Herrler T, et al. Distinct populations of cancer stem cells determine tumor growth and metastatic activity in human pancreatic cancer. Cell Stem Cell. 2007;1(3):313-323.	1252
10	Neoptolemos JP, Stocken DD, Friess H, et al. A randomized trial of chemoradiotherapy and chemotherapy after resection of pancreatic cancer. N Engl J Med. 2004;350(12):1200-1210.	1133
11	Olive KP, Jacobetz MA, Davidson CJ, et al. Inhibition of Hedgehog signaling enhances delivery of chemotherapy in a mouse model of pancreatic cancer. Science. 2009;324(5933):1457-1461.	1084
12	Li D, Xie K, Wolff R, et al. Pancreatic cancer. Lancet. 2004;363(9414):1049-57.	1013
13	Caldas C, Hahn SA, da Costa LT, et al. Frequent somatic mutations and homozygous deletions of the p16 (MTS1) gene in pancreatic adenocarcinoma. Nat Genet. 1994;8(1):27-32.	989
14	Oettle H, Post S, Neuhaus P, et al. Adjuvant chemotherapy with gemcitabine vs observation in patients undergoing curative-intent resection of pancreatic cancer - A Randomized controlled trial. JAMA. 2007;297(3):267-277.	978
15	Hidalgo M. Pancreatic cancer. N Engl J Med. 2010;362(17):1605-1617.	951
16	Lowenfels AB, Maisonneuve P, Cavallini G, et al. Pancreatitis and the risk of pancreatic cancer. N Engl J Med. 1993;328(20):1433-1437.	948
17	Thayer SP, di Magliano MP, Heiser PW, et al. Hedgehog is an early and late mediator of pancreatic cancer tumorigenesis. Nature. 2003;425(6960):851-856.	917
18	Hingorani SR, Petricoin EF, Maitra A, et al. Preinvasive and invasive ductal pancreatic cancer and its early detection in the mouse. Cancer Cell. 2003;4(6):437-435.	906
19	Liyanage UK, Moore TT, Joo HG, et al. Prevalence of regulatory T cells is increased in peripheral blood and tumor microenvironment of patients with pancreas or breast adenocarcinoma. J Immunol. 2002;169(5):2756-2761.	866
20	Sohn TA, Yeo CJ, Cameron JL, et al. Resected adenocarcinoma of the pancreas-616 patients: results, outcomes, and prognostic indicators. J Gastrointest Surg. 2000;4(6):567-579.	844
21	Yachida S, Jones S, Bozic I, et al. Distant metastasis occurs late during the genetic evolution of pancreatic cancer. Nature. 2010;467(7319):1114-1117.	828
22	Kalser MH, Ellenberg SS. Pancreatic cancer. Adjuvant combined radiation and chemotherapy following curative resection. Arch Surg. 1985;120(8):899-903.	738
23	Yeo CJ, Cameron JL, Lillemoe KD, et al. Pancreaticoduodenectomy for cancer of the head of the pancreas. 201 patients. Ann Surg. 1995;221(6):721-731.	702
24	Moertel CG, Frytak S, Hahn RG, et al. Therapy of locally unresectable pancreatic carcinoma: a randomized comparison of high dose (6000 rads) radiation alone, moderate dose radiation (4000 rads + 5-fluorouracil), and high dose radiation + 5-fluorouracil: The Gastrointestinal Tumor Study Group. Cancer. 1981;48(8):1705-1710.	699
25	Von Hoff DD, Ervin T, Arena FP, et al. Increased survival in pancreatic cancer with nab-paclitaxel plus gemcitabine. N Engl J Med. 2013;369(18):1691-1703.	691
26	Klinkenbijl JH, Jeekel J, Sahmoud T, et al. Adjuvant radiotherapy and 5-fluorouracil after curative resection of cancer of the pancreas and periampullary region: phase III trial of the EORTC gastrointestinal tract cancer cooperative group. Ann Surg. 1999;230(6):776-782.	677
27	Hingorani SR, Wang L, Multani AS, et al. Trp53R172H and KrasG12D cooperate to promote chromosomal instability and widely metastatic pancreatic ductal adenocarcinoma in mice. Cancer Cell. 2005;7(5):469-483.	647
28	Bloomston M, Frankel WL, Petrocca F, et al. MicroRNA expression patterns to differentiate pancreatic adenocarcinoma from normal pancreas and chronic pancreatitis. JAMA. 2007;297(17):1901-1908.	639
29	Winter JM, Cameron JL, Campbell KA, et al. 1423 pancreaticoduodenectomies for pancreatic cancer: A single-institution experience. J Gastrointest Surg. 2006;10(9):1199-1210.	633
30	Bardeesy N, DePinho RA. Pancreatic cancer biology and genetics. Nat Rev Cancer. 2002;2(12):897-909.	626
31	Louvet C, Labianca R, Hammel P, et al. Gemcitabine in combination with oxaliplatin compared with gemcitabine alone in locally advanced or metastatic pancreatic cancer: results of a GERCOR and GISCAD phase III trial. J Clin Oncol. 2005;23(15):3509-3516.	576
32	Cheng JQ, Ruggeri B, Klein WM, et al. Amplification of AKT2 in human pancreatic cells and inhibition of AKT2 expression and tumorigenicity by antisense RNA. Proc Natl Acad Sci U S A. 1996;93(8):3636-3641.	567
33	Tucker ON, Dannenberg AJ, Yang EK, et al. Cyclooxygenase-2 expression is up-regulated in human pancreatic cancer. Cancer Res. 1999;59(5):987-990.	566
34	Lee EJ, Gusev Y, Jiang J, et al. Expression profiling identifies microRNA signature in pancreatic cancer. Int J Cancer. 2007;120(5):1046-1054.	558
35	Geer RJ, Brennan MF. Prognostic indicators for survival after resection of pancreatic adenocarcinoma. Am J Surg. 1993;165(1):68-72.	557
36	Magnani JL, Steplewski Z, Koprowski H, et al. Identification of the gastrointestinal and pancreatic cancer-associated antigen detected by monoclonal antibody 19-9 in the sera of patients as a mucin. Cancer Res. 1983;43(11):5489-5492.	548
37	Vincent A, Herman J, Schulick R, et al. Pancreatic cancer. Lancet. 2011;378(9791):607-620.	542
38	Gudjonsson B. Cancer of the pancreas. 50 years of surgery. Cancer. 1987;60(9):2284-2303.	538
39	Hruban RH, van Mansfeld AD, Offerhaus GJ, et al. K-ras oncogene activation in adenocarcinoma of the human pancreas. A study of 82 carcinomas using a combination of mutant-enriched polymerase chain reaction analysis and allele-specific oligonucleotide hybridization. Am J Pathol. 1993;143(2):545-554.	530
40	Campbell PJ, Yachida S, Mudie LJ, et al. The patterns and dynamics of genomic instability in metastatic pancreatic cancer. Nature. 2010;467(7319):1109-1113.	523
41	Hruban RH, Takaori K, Klimstra DS, et al. An illustrated consensus on the classification of pancreatic intraepithelial neoplasia and intraductal papillary mucinous neoplasms. Am J Surg Pathol. 2004;28(8):977-987.	506
42	Smit VT, Boot AJ, Smits AM, et al. KRAS codon 12 mutations occur very frequently in pancreatic adenocarcinomas. Nucleic Acids Res. 1988;16(16):7773-7782.	504
43	Lowenfels AB, Maisonneuve P, DiMagno EP, et al. Hereditary pancreatitis and the risk of pancreatic cancer. International Hereditary Pancreatitis Study Group. J Natl Cancer Inst. 1997;89(6):442-446.	503
44	Wang W, Abbruzzese JL, Evans DB, et al. The nuclear factor-kappa B RelA transcription factor is constitutively activated in human pancreatic adenocarcinoma cells. Clin Cancer Res. 1999;5(1):119-127.	501
45	Huxley R, Ansary-Moghaddam A, Berrington de González A, et al. Type-II diabetes and pancreatic cancer: a meta-analysis of 36 studies. Br J Cancer. 2005;92(11):2076-2083.	497
46	Han HJ, Yanagisawa A, Kato Y, et al. Genetic instability in pancreatic cancer and poorly differentiated type of gastric cancer. Cancer Res. 1993;53(21):5087-5089.	494
47	Hezel AF, Kimmelman AC, Stanger BZ, et al. Genetics and biology of pancreatic ductal adenocarcinoma. Genes Dev. 2006;20(10):1218-1249.	491
48	Neoptolemos JP, Dunn JA, Stocken DD, et al. Adjuvant chemoradiotherapy and chemotherapy in resectable pancreatic cancer: a randomised controlled trial. Lancet. 2001;358(9293):1576-1585.	489
49	Sohn TA, Yeo CJ, Cameron JL, et al. Intraductal papillary mucinous neoplasms of the pancreas: an updated experience. Ann Surg. 2004;239(6):788-797.	482
50	Glimelius B, Hoffman K, Sjödén PO, et al. Chemotherapy improves survival and quality of life in advanced pancreatic and biliary cancer. Ann Oncol. 1996;7(6):593-600.	481
51	Dhillon N, Aggarwal BB, Newman RA, et al. Phase II trial of curcumin in patients with advanced pancreatic cancer. Clin Cancer Res. 2008;14(14):4491-4499.	479
52	Rozenblum E, Schutte M, Goggins M, et al. Tumor-suppressive pathways in pancreatic carcinoma. Cancer Res. 1997;57(9):1731-1734.	478
53	Berlin JD, Catalano P, Thomas JP, et al. Phase III study of gemcitabine in combination with fluorouracil versus gemcitabine alone in patients with advanced pancreatic carcinoma: Eastern Cooperative Oncology Group Trial E2297. J Clin Oncol. 2002;20(15):3270-3275.	473
54	Nitecki SS, Sarr MG, Colby TV, et al. Long-term survival after resection for ductal adenocarcinoma of the pancreas. Is it really improving? Ann Surg. 1995;221(1):59-66.	472
54	Aguirre AJ, Bardeesy N, Sinha M, et al. Activated Kras and Ink4a/Arf deficiency cooperate to produce metastatic pancreatic ductal adenocarcinoma. Genes Dev. 2003;17(24):3112-3126.	472
56	Conlon KC, Klimstra DS, Brennan MF. Long-term survival after curative resection for pancreatic ductal adenocarcinoma. Clinicopathologic analysis of 5-year survivors. Ann Surg. 1996;223(3):273-279.	471
57	Rothenberg ML, Moore MJ, Cripps MC, et al. A phase II trial of gemcitabine in patients with 5-FU-refractory pancreas cancer. Ann Oncol. 1996;7(4):347-353.	461
58	Sener SF, Fremgen A, Menck HR, et al. Pancreatic cancer: a report of treatment and survival trends for 100,313 patients diagnosed from 1985-1995, using the National Cancer Database. J Am Coll Surg. 1999;189(1):1-7.	458
59	Beatty GL, Chiorean EG, Fishman MP, et al. CD40 agonists alter tumor stroma and show efficacy against pancreatic carcinoma in mice and humans. Science. 2011;331(6024):1612-1616.	457
60	Rhim AD, Mirek ET, Aiello NM, et al. EMT and dissemination precede pancreatic tumor formation. Cell. 2012;148(1-2):349-361.	456
61	Cameron JL, Crist DW, Sitzmann JV, et al. Factors influencing survival after pancreaticoduodenectomy for pancreatic cancer. Am J Surg. 1991;161(1):120-124.	447
62	Roldo C, Missiaglia E, Hagan JP, et al. MicroRNA expression abnormalities in pancreatic endocrine and acinar tumors are associated with distinctive pathologic features and clinical behavior. J Clin Oncol. 2006;24(29):4677-4684.	445
63	Yeo CJ, Abrams RA, Grochow LB, et al. Pancreaticoduodenectomy for pancreatic adenocarcinoma: postoperative adjuvant chemoradiation improves survival. A prospective, single-institution experience. Ann Surg. 1997;225(5):621-633.	439
64	Van Cutsem E, van de Velde H, Karasek P, et al. Phase III trial of gemcitabine plus tipifarnib compared with gemcitabine plus placebo in advanced pancreatic cancer. J Clin Oncol. 2004;22(8):1430-1438.	438
65	Pedrazzoli S, DiCarlo V, Dionigi R, et al. Standard versus extended lymphadenectomy associated with pancreatoduodenectomy in the surgical treatment of adenocarcinoma of the head of the pancreas: a multicenter, prospective, randomized study. Ann Surg. 1998;228(4):508-517.	432
66	Korc M, Chandrasekar B, Yamanaka Y, et al. Overexpression of the epidermal growth factor receptor in human pancreatic cancer is associated with concomitant increases in the levels of epidermal growth factor and transforming growth factor alpha. J Clin Invest. 1992;90(4):1352-1360.	422
67	Hruban RH, Goggins M, Parsons J, et al. Progression model for pancreatic cancer. Clin Cancer Res. 2000;6(8):2969-2972.	417
68	Feldmann G, Dhara S, Fendrich V, et al. Blockade of hedgehog signaling inhibits pancreatic cancer invasion and metastases: a new paradigm for combination therapy in solid cancers. Cancer Res. 2007;67(5):2187-2196.	416
69	Everhart J, Wright D. Diabetes mellitus as a risk factor for pancreatic cancer. A meta-analysis. JAMA. 1995;273(20):1605-1609.	414
70	Wagner M, Redaelli C, Lietz M, et al. Curative resection is the single most important factor determining outcome in patients with pancreatic adenocarcinoma. Br J Surg. 2004;91(5):586-594.	412
71	Friess H, Yamanaka Y, Büchler M, et al. Enhanced expression of transforming growth factor beta isoforms in pancreatic cancer correlates with decreased survival. Gastroenterology. 1993;105(6):1846-1856.	410
72	Warshaw AL, Gu ZY, Wittenberg J, et al. Preoperative staging and assessment of resectability of pancreatic cancer. Arch Surg. 1990;125(2):230-233.	408
73	Goggins M, Schutte M, Lu J, et al. Germline BRCA2 gene mutations in patients with apparently sporadic pancreatic carcinomas. Cancer Res. 1996;56(23):5360-5364.	405
74	Goldstein AM, Fraser MC, Struewing JP, et al. Increased risk of pancreatic cancer in melanoma-prone kindreds with p16INK4 mutations. N Engl J Med. 1995;333(15):970-974.	400
75	Chang KJ, Nguyen P, Erickson RA, et al. The clinical utility of endoscopic ultrasound-guided fine-needle aspiration in the diagnosis and staging of pancreatic carcinoma. Gastrointest Endosc. 1997;45(5):387-393.	395
76	Schutte M, Hruban RH, Geradts J, et al. Abrogation of the Rb/p16 tumor-suppressive pathway in virtually all pancreatic carcinomas. Cancer Res. 1997;57(15):3126-3130.	393
77	Rösch T, Braig C, Gain T, et al. Staging of pancreatic and ampullary carcinoma by endoscopic ultrasonography. Comparison with conventional sonography, computed tomography, and angiography. Gastroenterology. 1992;102(1):188-199.	391
78	Bruns CJ, Solorzano CC, Harbison MT, et al. Blockade of the epidermal growth factor receptor signaling by a novel tyrosine kinase inhibitor leads to apoptosis of endothelial cells and therapy of human pancreatic carcinoma. Cancer Res. 2000;60(11):2926-2935.	387
79	Lan MS, Batra SK, Qi WN, et al. Cloning and sequencing of a human pancreatic tumor mucin cDNA. J Biol Chem. 1990;265(25):15294-15299.	384
80	MacMahon B, Yen S, Trichopoulos D, et al. Coffee and cancer of the pancreas. N Engl J Med. 1981;304(11):630-633.	383
81	Lieberman MD, Kilburn H, Lindsey M, et al. Relation of perioperative deaths to hospital volume among patients undergoing pancreatic resection for malignancy. Ann Surg. 1995;222(5):638-645.	382
82	Hwang RF, Moore T, Arumugam T, et al. Cancer-associated stromal fibroblasts promote pancreatic tumor progression. Cancer Res. 2008;68(3):918-926.	378
83	Rinehart J, Adjei AA, Lorusso PM, et al. Multicenter phase II study of the oral MEK inhibitor, CI-1040, in patients with advanced non-small-cell lung, breast, colon, and pancreatic cancer. J Clin Oncol. 2004;22(22):4456-4462.	376
84	Regine WF, Winter KA, Abrams RA, et al. Fluorouracil vs gemcitabine chemotherapy before and after fluorouracil-based chemoradiation following resection of pancreatic adenocarcinoma: a randomized controlled trial. JAMA. 2008;299(9):1019-1026.	371
85	Bramhall SR, Allum WH, Jones AG, et al. Treatment and survival in 13,560 patients with pancreatic cancer, and incidence of the disease, in the West Midlands: an epidemiological study. Br J Surg. 1995;82(1):111-115.	364
85	Heinemann V, Quietzsch D, Gieseler F, et al. Randomized phase III trial of gemcitabine plus cisplatin compared with gemcitabine alone in advanced pancreatic cancer. J Clin Oncol. 2006;24(24):3946-3952.	364
87	Tada M, Omata M, Kawai S, et al. Detection of ras gene mutations in pancreatic juice and peripheral blood of patients with pancreatic adenocarcinoma. Cancer Res. 1993;53(11):2472-2474.	361
88	Cullinan SA, Moertel CG, Fleming TR, et al. A comparison of three chemotherapeutic regimens in the treatment of advanced pancreatic and gastric carcinoma. Fluorouracil vs fluorouracil and doxorubicin vs fluorouracil, doxorubicin, and mitomycin. JAMA. 1985;253(14):2061-2067.	353
89	Logsdon CD, Simeone DM, Binkley C, et al. Molecular profiling of pancreatic adenocarcinoma and chronic pancreatitis identifies multiple genes differentially regulated in pancreatic cancer. Cancer Res. 2003;63(10):2649-2457.	349
90	Barton CM, Staddon SL, Hughes CM, et al. Abnormalities of the p53 tumour suppressor gene in human pancreatic cancer. Br J Cancer. 1991;64(6):1076-1082.	347
91	Casper ES, Green MR, Kelsen DP, et al. Phase II trial of gemcitabine (2,2′-difluorodeoxycytidine) in patients with adenocarcinoma of the pancreas. Invest New Drugs. 1994;12(1):29-34.	345
91	Mack TM, Yu MC, Hanisch R, et al. Pancreas cancer and smoking, beverage consumption, and past medical history. J Natl Cancer Inst. 1986;76(1):49-60.	345
93	Crile G Jr. The advantages of bypass operations over radical pancreatoduodenectomy in the treatment of pancreatic carcinoma. Surg Gynecol Obstet. 1970;130(6):1049-1053.	341
93	Yamanaka Y, Friess H, Kobrin MS, et al. Coexpression of epidermal growth factor receptor and ligands in human pancreatic cancer is associated with enhanced tumor aggressiveness. Anticancer Res. 1993;13(3):565-569.	341
95	Spitz FR, Abbruzzese JL, Lee JE, et al. Preoperative and postoperative chemoradiation strategies in patients treated with pancreaticoduodenectomy for adenocarcinoma of the pancreas. J Clin Oncol. 1997;15(3):928-937.	340
96	Sosa JA, Bowman HM, Gordon TA, et al. Importance of hospital volume in the overall management of pancreatic cancer. Ann Surg. 1998;228(3):429-438.	339
97	Redston MS, Caldas C, Seymour AB, et al. p53 mutations in pancreatic carcinoma and evidence of common involvement of homocopolymer tracts in DNA microdeletions. Cancer Res. 1994;54(11):3025-3033.	337
98	Pellegata NS, Sessa F, Renault B, et al. K-ras and p53 gene mutations in pancreatic cancer: ductal and nonductal tumors progress through different genetic lesions. Cancer Res. 1994;54(6):1556-1560.	334
99	Liebow C, Reilly C, Serrano M, et al. Somatostatin analogues inhibit growth of pancreatic cancer by stimulating tyrosine phosphatase. Proc Natl Acad Sci U S A. 1989;86(6):2003-2007.	333
100	Douglass HO. Further evidence of effective adjuvant combined radiation and chemotherapy following curative resection of pancreatic cancer. Gastrointestinal Tumor Study Group. Cancer. 1987;59(12):2006-2010.	330

**Table 3:** 10-year citations of the top-cited articles in pancreas cancer research

	Number of papers	Total citations	10-year citations	10-year/total (%)
1970–1979	1	341	70	20.5
1980–1989	12	7629	3145	41.2
1990–1999	42	22,971	11,434	49.8
2000–2005	21	12826	8718	68.0

**Fig. 1:**
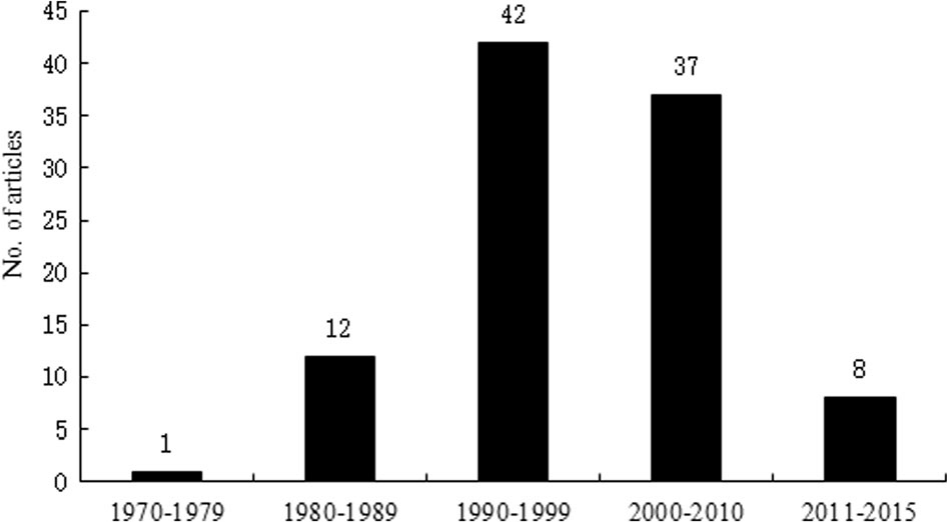
The numbers of classic citations in the past decades

The top 100 classic citations were published from 1970 to 2013. The decade of 1990–1999 produced the most classic citations with 42 ones, followed by 37 ones from 2000 to 2009 (Fig. 1). The most classic papers published were 7 ones in 1993, 1997, 2004, and 2007.

The top-cited articles were published in 37 high-impact journals (Table 4), led by *Cancer Research* (15 articles), followed by *Journal of Clinical Oncology* (9), *Annals of Surgery* (9), and *New England Journal of Medicine* (8).

**Table 4:** Journals in which the top 100 cited articles were published

Rank	Journals	No. of articles
1	*Cancer Research*	15
2	*Journal of Clinical Oncology*	9
2	*Annals of Surgery*	9
4	*New England Journal of Medicine*	8
5	*Journal of the American Medical Association*	5
6	*Science*	4
7	*Nature*	3
7	*Lancet*	3
7	*Cancer*	3
10	*Proceedings of the National Academy of Sciences*	2
10	*Journal of the National Cancer Institute*	2
10	*Journal of Gastrointestinal Surgery*	2
10	*Genes & Development*	2
10	*Gastroenterology*	2
10	*Clinical Cancer Research*	2
10	*Cell*	2
10	*Cancer Cell*	2
10	*British Journal of Surgery*	2
10	*British Journal of Cancer*	2
10	*Archives of Surgery*	2
10	*Annals of Oncology*	2
10	*American Journal of Surgery*	2
23	*Surgery, gynecology & obstetrics*	1
23	*Nucleic Acids Research*	1
23	*Nature Reviews Cancer*	1
23	*Nature Genetics*	1
23	*Journal of Immunology*	1
23	*Journal of Clinical Investigation*	1
23	*Journal of Biological Chemistry*	1
23	*Journal of the American College of Surgeons*	1
23	*Investigational New Drugs*	1
23	*International Journal of Cancer*	1
23	*Gastrointestinal Endoscopy*	1
23	*Cell Stem Cell*	1
23	*Anticancer Research*	1
23	*American Journal of Surgical Pathology*	1
23	*American Journal of Pathology*	1

The 100 top-cited articles came from 11 countries, with the USA producing 79 articles, followed by UK with 6 ones, and Germany with 4 ones (Table 5). Altogether, 51 institutions produced these 100 top-cited papers. Among them, 12 institutions produced 2 or more of the top-cited papers (Table 6), led by Johns Hopkins University (20 articles), followed by the University of Texas (7 articles), and Harvard University (7 articles).

**Table 5:** Countries of origin of the top 100 cited articles

Rank	Countries	No. of articles
1	USA	79
2	UK	6
3	Germany	4
4	France	2
4	Netherlands	2
6	Australia	1
6	Belgium	1
6	Canada	1
6	Italy	1
6	Japan	1
6	Sweden	1

**Table 6:** Institutions of origin with two or more top-cited articles

Rank	Institutions	No. of articles
1	Johns Hopkins University	20
2	University of Texas	7
2	Harvard University	7
4	Memorial Sloan-Kettering Cancer Center	4
4	University of California, Irvine	4
4	University of Pennsylvania	4
7	Mayo Clinic Rochester	3
7	Ohio State University	3
9	Liverpool University	2
9	National Institute of Arthritis, Diabetes, and Digestive and Kidney Diseases, NIH	2
9	New York Medical College	2
9	University of Michigan	2

In the 100 top-cited articles, 3 ones were authored by a single author and 5 ones by 2 authors. Twenty-seven persons authored two or more of the top citations. Table 6 presents a list of these “most frequent authors,” which is led by Kern SE who authored 6 classic papers and Yeo CJ who authored 5 classic papers.

Of the 100 top-cited papers, 40 were observational studies, 36 dealt with basic science, and 14 were randomized controlled trials (RCT). The other 9 papers were 7 review articles, 1 conference consensus, and 1 meta-analysis (Table 7). Among the 56 clinical studies (40 observational studies, 14 RCTs, and 2 meta-analyses), 39 were about treatment (including 14 chemotherapy, 9 chemoradiotherapy, and 16 surgery), 9 were about diagnosis (2 radiographic and 8 laboratory), and 7 were about epidemiology (Table 8).

**Table 7:** Most common authors of the top 100 cited articles

Rank	Author	Corresponding	First	Second	Total
1	Kern SE	6	–	–	6
2	Yeo CJ	4	1	–	5
3	Hruban RH	2	1	1	4
4	Cameron JL	2	–	2	4
4	Schutte M	–	1	2	3
4	Neoptolemos JP	2	1	–	3
4	Moore MJ	1	–	2	3
4	Korc M	3	–	–	3
4	Goggins M	1	1	1	3
4	DePinho RA	3	–	–	3
4	Brennan MF	2	–	1	3
4	Abbruzzese JL	1	–	2	3
13	Yamanaka Y	–	1	1	2
13	Yachida S	1	–	1	2
13	Warshaw AL	1	1	–	2
13	Von Hoff DD	2	–	–	2
13	Tuveson DA	2	–	–	2
13	Sohn TA	–	1	1	2
13	Simeone DM	1	–	1	2
13	Moertel CG	1	–	1	2
13	Maisonneuve P	–	–	2	2
13	Lowenfels AB	2	–	–	2
13	Jones S	–	1	1	2
13	Hingorani SR	1	1	–	2
13	Friess H	–	1	1	2
13	Caldas C	–	1	1	2
13	Bardeesy N	–	1	1	2

**Table 8:** Study design of the top 100 cited articles

Study design	No. of articles
Observational study	40
Basic science	36
Randomized controlled trial	14
Review article	7
Conference consensus	1
Meta-analysis	2

## Discussion

Bibliometric analysis of most frequently cited articles and the journals in which they appear serves several purposes. It identifies and emphasizes the impact of the work of our colleagues and predecessors, recognizes key advances in pancreatic cancer research, and adds useful perspective on historical developments in this specialty. The use of citation analysis to examine the pancreatic cancer research literature also reveals quantitative information about authors, topics, and journals that is helpful in identifying classic works and high-impact journals. As far as we know, this is the first bibliometric analysis on the top citations in the field of pancreatic cancer research.

Although it is very difficult to provide a detailed analysis of all the 100 top citations, some interesting observations could be made about the top 10. These 10 classic citations revealed major advances in PC research and a number of hot topics in the past five decades. The leading article by Burris HA 3rd reported the first large-size RCT on gemcitabine in advanced PC which may be the most important advance in the field of medical treatment of PC research in the past decades. At position 2, Moore MJ firstly described a large-size clinical RCT of erlotinib plus gemcitabine compared with gemcitabine alone in patients with advanced PC. At position 3 and 5, core signaling pathways revealed by global genomic analyses and mutant c-K-ras genes were reported. At position 4 and 9, identification of pancreatic cancer stem cells and the effect on tumor growth and metastatic activity of distinct populations of pancreatic cancer stem cells were described. At position 6, Guillemin R founded that growth hormone-releasing factor from a human pancreatic tumor could cause acromegaly. At position 7, Conroy T conducted a RCT of FOLFIRINOX versus gemcitabine for metastatic pancreatic cancer. At position 8, Warshaw AL reviewed the advances of pancreatic cancer. At position 10, Neoptolemos JP compared chemoradiotherapy and chemotherapy after resection of pancreatic cancer in a RCT.

Most of the classic articles are still regularly cited now. Time has much effect on an article’s citation, because of that an article’s citations depend on its publication time, as citations accumulate over time. Hence, the group of the highly cited articles could be dominated by the earliest papers. However, the decade 1990–1999 and 2000–2009 produced the most classic citations with 42 and 37 ones, respectively, which indicated that much more advances were achieved in these two decades than the others, such as gemcitabine. Besides, in the most recent years, many new journals were developed, and therefore, the number of published articles has grown rapidly and more references are cited.

There were 15, 9, and 9 classic articles published in *Cancer Research*, *Journal of Clinical Oncology*, and *Annals of Surgery*, respectively, which showed that these three journals were the leading ones in the speciality of PC research. *New England Journal of Medicine*, *Journal of the American Medical Association*, and *Lancet* were the most famous general medical journals, and 16 articles were published in these three journals. The fact that papers on PC research are regularly published on these journals documents that PC is an important topic and health issue to humans.

The finding that most top-cited articles originated from the USA is anticipatory and is consistent with the origins of the citation classics in other fields [2–5]. Furthermore, 11 of the 12 top productive institutions lie in USA. These findings confirm the USA’s overwhelming impact on PC research because of its large population and abundant financial supports to the scientific community.

The list of the most frequent authors on PC research just gave a sample of some of the best recognized scientists in PC research. It was not surprising that not all famous scientists in PC research were mentioned, which was consistent with the previous studies on top citation classics [2–5]. The editors may consider inviting these researchers to submit subsequent manuscripts or reviews. In many cross-discipline studies of highly-cited articles, reviews usually predominate [10]. In this study, there were 7 reviews in the top 100 cited articles.

Some of the articles reported are not original research articles, except the reviews. They are highly cited because they are “opinion” articles, that for several reasons became classic in the field of pancreatology. For example, the article from Crile keeps being cited since it is an example of the so-called nihilistic approach to pancreatic cancer. Although they are not original research articles, they are also very important to the advance of pancreatic cancer, and thus, they are constantly cited.

In this literature analysis, by searching for “pancreatic cancer research”, the top articles were mainly on oncology and basic cancer research. This paper maybe just provides a good view of the medical part of the research world and does not fully reflect the great contribution to this field from surgeons. Some important surgical advances were achieved before several decades, when not many medical journals were published and the number of citations could not fully reflect the influence.

## Conclusions

The top-cited articles identify topics, authors, and institutions that contributed to major advances in the speciality of PC research. This analysis allows for the recognition of major advances in PC research and gives a historic perspective on the progress of PC research.

## Abbreviations

PC: Pancreatic cancer
RCT: Randomized controlled trials
